# ABO Blood Group Incompatibility Protects Against SARS-CoV-2 Transmission

**DOI:** 10.3389/fmicb.2021.799519

**Published:** 2022-01-05

**Authors:** Rachida Boukhari, Adrien Breiman, Jennifer Jazat, Nathalie Ruvoën-Clouet, Salima Martinez, Anne Damais-Cepitelli, Catherine Le Niger, Isabelle Devie-Hubert, Fanny Penasse, Dominique Mauriere, Véronique Sébille, Antoine Dürrbach, Jacques Le Pendu

**Affiliations:** ^1^CHU de Nantes, Nantes, France; ^2^Université de Nantes, Inserm, CRCINA, Nantes, France; ^3^Oniris, Ecole Nationale Vétérinaire, Agroalimentaire et de l’Alimentation, Nantes, France; ^4^Unité d’hémovigilance, CHU de Toulouse, Toulouse, France; ^5^Hémovigilance, Groupe Hospitalier du Havre, Le Havre, France; ^6^CHU de Brest, Brest, France; ^7^Institut Godinot, CLCC, Reims, France; ^8^Service Pharmacie, CH Bar sur Aube, Bar sur Aube, France; ^9^Service hémovigilance, CHR Metz-Thionville, Metz, France; ^10^Methodology and Biostatitics Unit, CHU de Nantes, Nantes, France; ^11^Université de Nantes, Université de Tours, Inserm, SPHERE U1246, Nantes, France; ^12^Service Néphrologie-Dialyse-Transplantation, CHU Henri Mondor, Inserm, UMR 1186, Institut Gustave Roussy, Université Paris Saclay, Créteil, France

**Keywords:** COVID-19, SARS-CoV-2 infection, ABO blood groups, incompatibility, genetic susceptibility and resistance

## Abstract

ABO blood groups appear to be associated with the risk of SARS-CoV-2 infection, but the underlying mechanisms and their real importance remain unclear. Two hypotheses have been proposed: ABO compatibility-dependence (neutralization by anti-ABO antibodies) and ABO-dependent intrinsic susceptibility (spike protein attachment to histo-blood group glycans). We tested the first hypothesis through an anonymous questionnaire addressed to hospital staff members. We estimated symptomatic secondary attack rates (SAR) for 333 index cases according to spouse ABO blood group compatibility. Incompatibility was associated with a lower SAR (28% vs. 47%; OR 0.43, 95% CI 0.27–0.69), but no ABO dependence was detected in compatible situations. For the second hypothesis, we detected no binding of recombinant SARS-CoV-2 RBD to blood group-containing glycans. Thus, although no intrinsic differences in susceptibility according to ABO blood type were detected, ABO incompatibility strongly decreased the risk of COVID-19 transmission, suggesting that anti-ABO antibodies contribute to virus neutralization.

## Introduction

**Figure 1:**
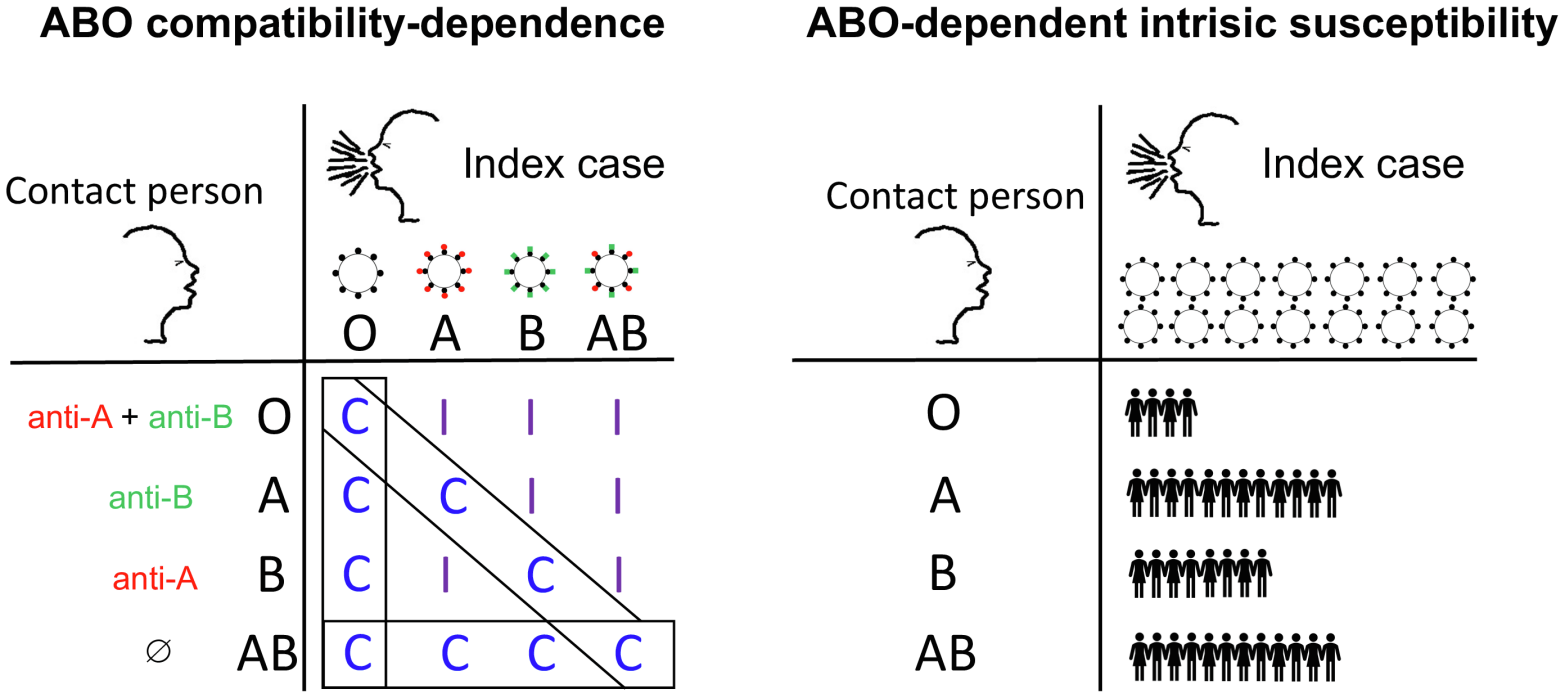
Proposed hypotheses to account for the reported impact of ABO phenotype on the risk of SARS-CoV-2 infection. Under the compatibility-dependence hypothesis (left), protection through virus neutralization is mediated by pre-existing natural anti-ABO antibodies that recognize blood group antigens carried by the virus envelope glycans. Index cases of blood groups A, B or AB excrete virions carrying the A antigen (red spikes), the B antigen (green spikes) or both. Contacts may have anti-A (red) and/or anti-B (green) antibodies able to neutralize the virus carrying the cognate antigen. Protection thus occurs only in situations of ABO incompatibility (I) between the index case and the contact. In the context of compatible encounters (C boxed), no effect of ABO phenotype would be expected. Under the ABO-dependent intrinsic susceptibility hypothesis (right), individuals of blood groups A, B and AB may be intrinsically more susceptible to infection than individuals of blood group O, regardless of the blood group of the person transmitting the virus. Thus, for the same numbers of virions excreted by the index case, the number of contact individuals infected depends on the ABO blood group of the contact. This difference in susceptibility may be due to a direct attachment of the virus spike protein to blood group type glycans (such as the A antigen), facilitating the infection process. Note that although the two hypotheses are not mutually exclusive, their expected consequences are very different. Thus, under the compatibility-dependence hypothesis, transmission rates in populations with a high blood group O frequency should be higher than those in populations in which this blood group is less frequent, because the frequency of compatible encounters is higher. Conversely, under the ABO-dependent intrinsic susceptibility hypothesis, the populations with the highest frequencies of blood group O should benefit from the lower susceptibility conferred by the O blood group.

Following an initial study in Wuhan and Shenzhen in China, early in the coronavirus disease 2019 (COVID-19) pandemic (Zhao et al., 2020), a large number of studies reported associations between ABO blood group and COVID-19. Most studies reported a lower risk of infection for people of blood group O than for those of non-O blood groups, with blood group A, in particular, associated with a higher risk (reviewed in Goel et al., 2021). Some discrepancies between studies appeared, but a recent updated meta-analysis concluded that individuals of blood group O were, indeed, less susceptible to SARS-CoV-2 infection than non-O individuals (Franchini et al., 2021). Overall, these studies suggest that the impact of ABO phenotype on SARS-CoV-2 transmission is modest. Nevertheless, the true impact of these phenotypes remains difficult to assess, as it may depend on the underlying mechanisms, the frequencies of the ABO blood groups in the population concerned, and the fraction of the population that has already been infected at the time of the study, as recently discussed (Le Pendu et al., 2021). Several pathophysiological mechanisms have been proposed to explain these associations between ABO blood type and SARS-CoV-2 infection (AbdelMassih et al., 2020; Goel et al., 2021; Zhang et al., 2021). SARS-CoV-2 replicates in respiratory tract cells that express A, B, or H(O) antigens according to the infected person’s ABO blood group; the corresponding host cells glycosyltransferases can act on nascent glycans of the viral envelope glycoproteins, which therefore will carry the epitopes. In addition, virions are carriers of a portion of the membrane of infected cells, thus the corresponding carbohydrate antigens would therefore be expected to be present on the excreted virion glycans (Deleers et al., 2021). Natural anti-A and anti-B antibodies present in ABO-incompatible virus recipients could accordingly play a role in the neutralization of these virions, either by blocking the interaction with ACE2, or by elimination through opsonization. Such mechanisms involving ABO blood group-related antigens have already been reported for several other enveloped viruses (Durrbach et al., 2007; Guillon et al., 2008; Galili, 2020). Regardless of the precise mechanism of neutralization, this role of anti-ABO antibodies has been described as “ABO interference” (Ellis, 2021). Alternatively, the receptor-binding domain (RBD) of the viral spike protein may act as a lectin, facilitating attachment to a blood group A epitope present on the respiratory and digestive epithelial cells of blood group A individuals, favoring infection and accounting for the higher susceptibility of blood group A individuals than of individuals of the other ABO types (Wu et al., 2021). Such a mechanism has already been reported for some strains of noroviruses and rotaviruses (Le Pendu and Ruvöen-Clouet, 2019), contributing to so-called “ABO-dependent intrinsic susceptibility” (Figure 1). The two types of potential mechanisms – ABO compatibility-dependence (or ABO interference) and ABO-dependent intrinsic susceptibility – although not mutually exclusive, have different consequences that may have blurred the results of early epidemiological studies. Here, we aimed to distinguish between these two major pathophysiological mechanisms, by analyzing the effects of ABO compatibility or incompatibility on the risk of SARS-CoV-2 transmission in a population of individuals of known ABO blood type with a high risk of transmission. We asked hospital staff members to complete a questionnaire to enable us to calculate the secondary attack rate (SAR) for transmission from COVID-19 index cases to their spouses according to the ABO compatibility/incompatibility of the potential transmission events. We also reassessed SARS-CoV-2 RBD binding to blood group A epitopes.

## Materials and Methods

### Study Design and Participant Recruitment

Hospital employees were asked to complete an anonymous questionnaire *via* the hospital’s weekly COVID-19 information letter. The study was conducted between April and July 2021. The questionnaire was accessible online with the WEPI online tool for epidemiologists and healthcare professionals. It comprised 11 items that are listed in Table 1.

**Table 1:** Content of the anonymous questionnaire.

Item	Response format
Region (French administrative region)?	List of 18 regions^*^
*Département* (French administrative subdivision)?	List of 100 départements^*^
Year of contamination?	2020/2021
Month of contamination?	List of 12 months^*^
First person contaminated?	You/Your partner
Do you share the same bedroom?	Yes/No
Have you had PCR-confirmed COVID-19?	Yes/No
Did your partner have PCR-confirmed COVID-19?	Yes/No
If you both got sick (or had a positive PCR test), how long was it between the first and the second person getting sick (or testing positive)?	3 < 8 d/>8 d/NA^†^
What is your ABO blood group?	A/B/O/AB
What is your partner’s ABO blood group?	A/B/O/AB

* *Multiple-choice list*.

† *Less than 3 days/between 3 and 8 days/more than 8 days/not available*.

The inclusion criteria were: PCR-confirmed COVID-19 in at least one of the individuals of the couple; if both partners had been ill, clear identification of the first individual infected; if both partners had been ill, symptom onset in the second partner affected within 8 days of symptom onset in the first partner. The ABO blood groups of both partners had to be known. The exclusion criteria were: the two partners not sharing the same bedroom; symptom onset in the second partner more than 8 days after that in the first partner.

A pilot study was conducted at Nantes University Hospital, in which 89 responses were obtained, 83 of which satisfied the inclusion criteria. COVID-19 transmission occurred in 22 couples (SAR: 26.5%). Based on the ABO frequencies in the French population,^1^ incompatible encounters, as defined in Figure 1, were expected to account for 34% of all encounters. With the initial hypothesis that anti-ABO antibodies, when present, in incompatible transmission events, would provide 50% protection, we estimated that we would need to recruit at least 300 couples to be able to detect such protection with 90% power, and a 5% type I error, assuming a 32% probability of COVID-19 transmission between ABO-compatible individuals, deduced from these assumptions.

### Outcomes

The primary outcome was the effect of ABO incompatibility on the risk of COVID-19 in the second partner. The secondary outcomes were the detection of a potential intrinsic susceptibility of non-O blood group individuals, and the replication of previous studies showing a lower risk of infection in blood group O individuals than in non-O blood group individuals.

### Data Analysis and Statistics

We identified instances of the following four categories from the questionnaire: ABO-compatible COVID-19 transmission; ABO-incompatible COVID-19 transmission; ABO-compatible absence of COVID-19 transmission; ABO-incompatible absence of COVID-19 transmission. Two-tailed Fisher’s exact tests or chi-squared tests were used for comparisons. A multivariable logistic regression model was used to assess the simultaneous effects of blood group and ABO incompatibility on the probability of COVID-19 transmission. Values of *p* ≤ 0.05 were considered as significant. Analyses were performed with Prism software version 8.4.3 and SAS statistical software (SAS 9.4 Institute, Cary, NC).

### Attachment of the SARS-CoV-2 Spike Protein RBD to Histo-Blood Group Antigens

HEK-293T cells were transiently transfected with a plasmid encoding the receptor-binding domain (RBD) of the SARS-CoV-2 spike protein fused to the Fc domain of a mouse IgG (obtained from Dr. Jianxun Qi, Chinese Academy of Sciences, Beijing). Crude cell supernatant was used as a source of the RBD-Fc fusion protein, because this protein was the major protein on gel electrophoresis.

ELISA plates (Maxisorp, Nunc, Thermo Fisher Scientific, Roskilde, Denmark) were coated with 1 μg/ml recombinant human ACE2, 10 μg/ml A type 1 hexasaccharide or H type 1 pentasaccharide coupled to human serum albumin (HSA), or boiled saliva samples from individuals of known ABO and secretor phenotypes diluted 1/1,000 as previously described (Khachou et al., 2020). The plates were washed three times with 0.05% Tween 20 in PBS and blocked with 5% BSA in PBS. The RBD-Fc fusion protein was added to the ACE2- and neoglycoconjugate-coated plates, or to the saliva coated plates, which were then incubated overnight at 4°C. The plates were washed and incubated with a horseradish peroxidase-conjugated anti-mouse IgG (Upima, Interchim, Montluçon, France) for 1 h at room temperature. Finally, the plates were incubated with the TMB substrate and reactions were stopped by adding 1 M phosphoric acid. Optical densities were read at 450 nm with a SPECTROstar nanospectrophotometer (BMG Labtech, Champigny-sur-Marne, France).

Flow cytometry experiments were performed using ACE2 stably-transfected HEK-293 cells and their non-transfected counterpart. Briefly, after being detached with PBS-EDTA, and resuspended in PBS-0.1% BSA, cells were incubated with the RBD-Fc-containing supernatant diluted ½ for 90 min at 4°C, followed by an FITC-labeled goat anti-mouse IgG(H + L) 1:200 (Beckman Coulter). Analysis was performed on a Celesta flow cytometer using the DIVA software (BD Biosciences).

Ethanol-fixed lung tissue sections from a blood group A and a blood group O secretor donor were obtained from the Nantes University Hospital Center for Biological Resources (approval no. DC-2011-1399). Immunohistochemistry was performed as previously described (Breiman et al., 2016). Briefly, following paraffin removal and blocking steps, sections were incubated overnight with the anti-A blood group monoclonal antibody ABO1 clone 9113D10 (Diagast, Loos, France) at a 1/10 dilution or with the RBD-Fc-containing supernatant diluted 1/2. The slides were washed and successively incubated with HRP-conjugated anti-mouse IgG (Uptima, Interchim, Montluçon, France), the Impact VIP substrate and methyl green counterstain (Vector Laboratories, Burlingame, CA, United States). They were then mounted and imaged with a nanozoomer slide-scanner (Hamamatsu Photonics, Massy, France).

## Results

### General Characteristics of the Cohort

The questionnaire yielded 387 responses. In 35 couples, the index case had a positive PCR test for SARS-CoV-2 but had remained asymptomatic. In these couples, three partners (8.6%) became symptomatic. As PCR-confirmed COVID-19 for primary and secondary cases was the only inclusion criterion met (not possible to determine the interval between the infections of the two partners), these couples were excluded from the study. For the remaining 352 couples, 19 slept in separate bedrooms or the symptoms of the secondary case appeared more than 8 days after those of the primary case. As these were exclusion criteria, the corresponding couples were also removed from the analysis, yielding a total of 333 couples for the study.

The ABO blood group frequencies for the 666 members of these 333 couples did not differ significantly from those in the French general population (42.9% A, 7.1% B, 46.1% O and 3.9% AB vs. 44.5% A, 9.1% B, 42.5% O, and 3.9% AB, respectively). Chi-squared analysis also showed that there was no significant difference in ABO blood group distribution between the index cases and the French general population, despite an apparently lower frequency of blood group O and a higher frequency of blood group A (Table 2).

**Table 2:** Distribution of ABO blood groups according to COVID-19 status in responding couples and in the French general population.

ABO blood group	Total	Index cases	COVID-19^+^^*^	COVID-19^−^^†^	French population^‡^
A	286 (42.9)	167 (50.2)	221 (47.6)	65 (32.2)	(44.5)
AB	26 (3.9)	13 (3.9)	20 (4.3)	6 (3.0)	(3.9)
B	47 (7.1)	21 (6.3)	31 (6.7)	16 (7.9)	(9.1)
O	307 (46.1)	132 (39.6)	192 (41.4)	115 (56.7)	(42.5)

* *The values are number (%) including index cases and secondary cases*.

† *Chi-squared comparison between the COVID-19^+^ and COVID-19^−^ groups, p = 0.0011*.

‡ *Data expressed as a % from https://www.ints.fr/SangTransfGrSanguin.aspx*.

Secondary cases occurred in 131 couples, yielding a secondary attack rate for COVID-19 between partners of 39.3% (Table 3). The SAR was significantly higher in this included group of couples with a symptomatic index case than in the 35 couples excluded because the index case was asymptomatic (Fisher’s test, *p* = 0.0002), consistent with the findings of earlier studies (Madewell et al., 2020).

**Table 3:** Effect of ABO compatibility on COVID-19 transmission within couples.

	All couples		Couples 2020		Couples 2021	
	Yes^*^	No	Yes	No	Yes	No
Total	131 (39.3)	202 (60.7)	76 (33.6)	150 (66.4)	55 (51.4)	52 (48.6)
Compatible	93	104	54	80	39	24
Incompatible	38	98	22	70	16	28

* *The values given are the numbers of couples in which secondary cases occurred (Yes) or did not occur (No)*.

### Effect of ABO Blood Group on Secondary Transmission

**Figure 2:**
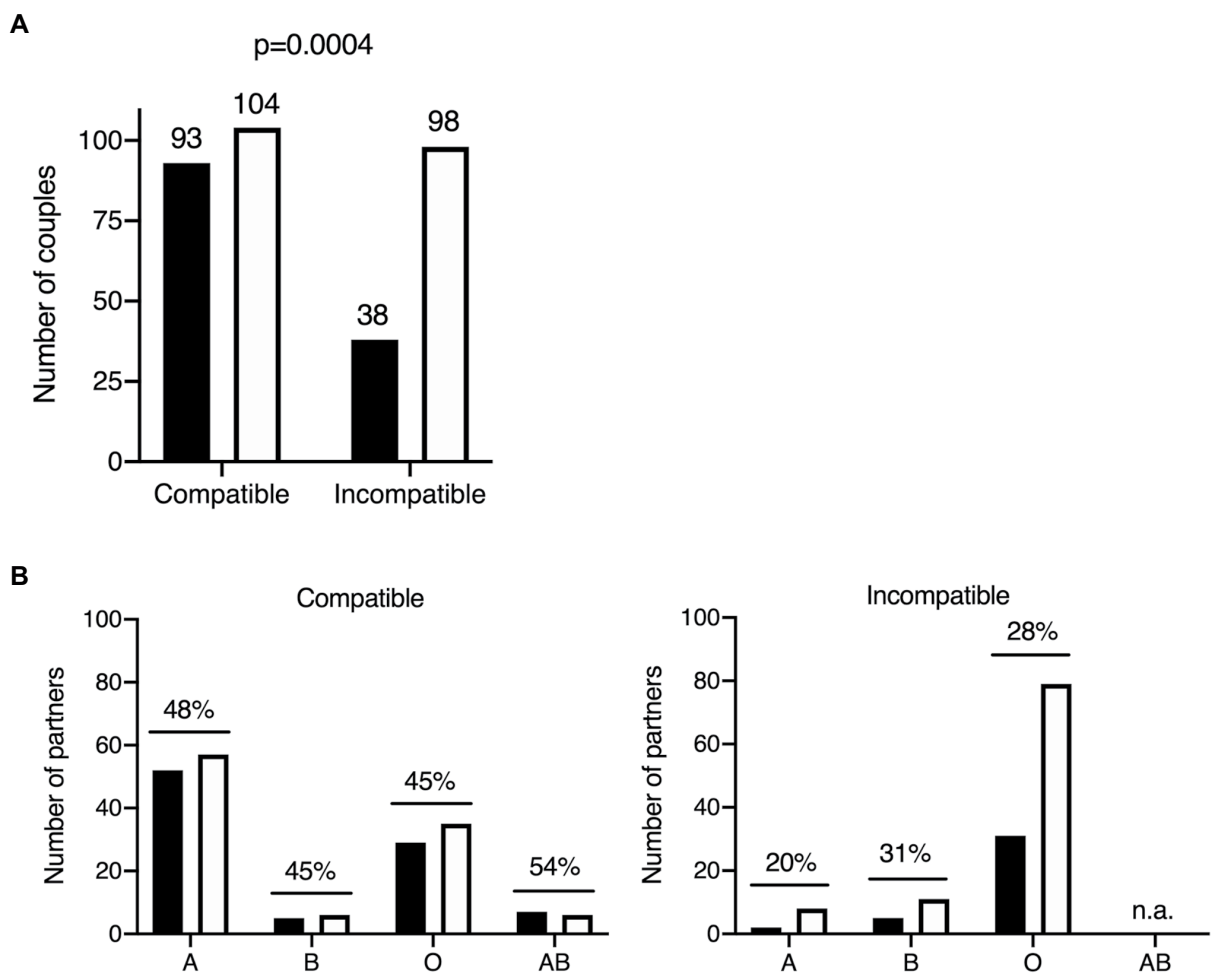
Effects of ABO blood group on COVID-19 transmission within the cohort of couples. Two-tailed Fisher’s exact test of COVID-19 transmission within couples according to ABO compatibility. The number of individuals in each group is indicated above the bar. Black bars: couples with disease transmission; white bars: couples without disease transmission **(A)**. ABO blood group distribution of the partners of COVID-19 primary cases in situations of ABO compatibility (left panel) and ABO incompatibility (right panel). Black bars: cases with COVID-19 transmission; white bars: cases without COVID-19 transmission. Secondary attack rates are shown above bars for each ABO type; n.a., not applicable because the AB blood type is always compatible (universal recipient; **B**).

COVID-19 transmission occurred between 93 ABO-compatible partners, but between only 38 ABO-incompatible partners. We found that 98 couples of the couples in which no transmission occurred were ABO-incompatible, whereas 104 were ABO-compatible (Table 3; Figure 2A). ABO incompatibility therefore appeared to be associated with a lower risk of symptomatic COVID-19 transmission (*p* = 0.0004; OR 0.43, 95% CI 0.27–0.69). The SAR was 47.2% for ABO-compatible couples, but only 27.9% for ABO-incompatible couples, corresponding to a 41% decrease.

The index cases of the cohort were infected between January 2020 and May 2021. As the vaccination of French hospital staff began in early 2021 and the alpha variant of SARS-CoV-2 became predominant during the first few months of 2021, we analyzed the data for 2020 and 2021 separately (Table 3). Transmission occurred in 76 of 226 couples in 2020 (SAR = 33.6%), and 55 of 107 couples in 2021 (SAR = 51.4%). The SAR in 2021 was significantly higher than that in 2020 (Fisher’s exact test *p* = 0.0026, OR 2.1, 95% CI 1.3–3.3). This is likely explained by the higher transmissibility of the alpha variant of SARS-CoV-2 that has become the dominant strain during the first few months of 2021, in comparison to the initial strain. The effect of ABO incompatibility was similar between the two periods (*p* = 0.015, OR 0.047, 95% CI 0.26–0.89 for 2020 and *p* = 0.011, OR 0.35, 95% CI 0.16–0.78 for 2021), indicating that differences in the epidemiological situation between the two periods did not affect the impact of ABO incompatibility.

We further investigated the impact of blood group on disease transmission, by classifying ABO-compatible and ABO-incompatible couples according to the ABO blood group of the second partner. The SAR was higher for ABO-compatible couples than for ABO-incompatible couples, regardless of the blood group of the second partner considered (Figure 2B). This suggests that all ABO blood groups are intrinsically equally susceptible to COVID-19 (logistic model *p* > 0.05 for the blood group effect and *p* = 0.0043 for the ABO incompatibility effect, OR 2.2, 95% CI 1.3–3.9). For virus transmission in an ABO-incompatible context, the SAR was lower regardless of ABO blood group (except for blood group AB, which, by definition, cannot be incompatible). In the Western European population, of which our French cohort is representative, blood group A individuals are more rarely in incompatible couples than individuals of blood groups O and B, owing to the relative frequencies of these phenotypes (Figure 2B).

We then compared ABO frequencies between the COVID-19-positive individuals of our cohort and COVID-19-negative individuals (Table 2). ABO frequencies in the COVID-19-positive subgroup were similar to those of the general population, with only a slightly higher frequency of blood group A and a slightly lower frequency of blood group O, neither of these differences being significant. However, the COVID-19-negative subgroup had a much higher frequency of blood group O and a much lower frequency of blood group A. A Fisher’s test comparison of the O and non-O groups showed that the frequency of blood group O was significantly higher in the COVID-19-negative subgroup than in the COVID-19-positive subgroup (*p* = 0.0003, OR 0.53, 95% CI 0.38–0.74), whereas a comparison of A and non-A blood groups showed a higher frequency of blood group A in the COVID-19-positive subgroup (*p* = 0.0002, OR 1.92, 95% CI 1.35–2.70).

### Attachment of the RBD to A or B Histo-Blood Group Antigens

**Figure 3:**
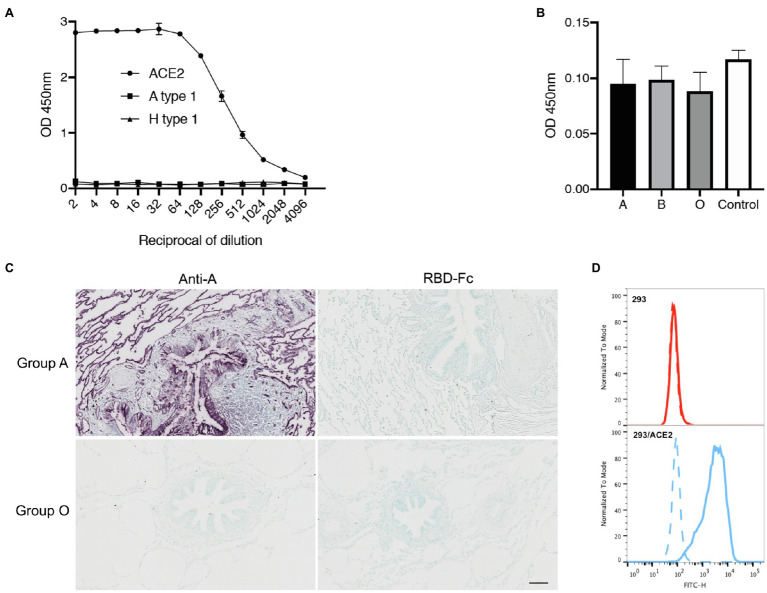
Assay of SARS-CoV-2 RBD binding to blood group A or B antigens. Binding of the RBD-Fc recombinant protein to ACE2 relative to that to the A type 1 hexasaccharide and the H type 1 pentasaccharide **(A)** and to saliva samples from 19 blood group A, 10 blood group B and 19 blood group O secretors **(B)** as determined by ELISA. The data shown are the OD values obtained in two independent experiments performed in duplicate. The negative control is the mean OD value obtained in the absence of saliva coating, for three independent plates. Lung tissue sections from a Secretor blood group A and a blood group O donor were incubated with either an anti-A blood group monoclonal antibody or the RBD-Fc fusion protein diluted 1/2 **(C)**. Scale bar: 100 μm. Flow cytometry detection of ACE2 on the surface of ACE2 transfected HEK-293 cells by the RBD-Fc construct. The negative control, in absence of fusion protein is shown by a dashed line, labeling of control HEK-293 cells and of ACE2-expressing HEK-293 cells are shown by red and blue histograms, respectively **(D)**.

It has been suggested that the attachment of the SARS-CoV-2 spike protein to A type 1 histo-blood group antigen *via* its RBD could account for the apparently higher risk of infection in individuals of blood group A (Wu et al., 2021). We found that the risk of COVID-19 transmission to blood group A individuals was no higher than that to members of other blood groups in situations of ABO compatibility, calling into question the ability of the SARS-CoV-2 RBD to attach to a blood group A structure. A type 1 is largely expressed on epithelial cells, whereas A type 2 is present on erythrocytes. The two structures differ in terms of the nature of the underlying glycan precursor (Galβ3GlcNAc vs. Galβ4GlcNAc). In our assay conditions, we detected no binding of the recombinant RBD-Fc chimeric protein to the A type 1 hexasaccharide, whereas strong binding to the well-known ACE2 receptor was observed (Figure 3A). Histo-blood group antigens with structures similar to those expressed in epithelia are present in salivary mucins. We therefore tested the ability of the RBD to attach to salivary mucins as a function of donor ABO blood group. Secretor-phenotype saliva samples were selected so as to ensure A, B, or H(O) histo-blood group antigen expression. No binding above background levels was observed, regardless of the donor ABO phenotype (Figure 3B). We then assessed the attachment of the SARS-CoV-2 recombinant RBD to the lung tissue of a blood group A and a blood group O donor. Strong expression of the A antigen on the lung tissue of the blood group A individual was confirmed, but, again, no binding of the RBD to the tissue sections was observed (Figure 3C). ACE2 is present on lung cells, so binding might have been anticipated regardless of blood group (A or O). Tissue processing destroys many protein epitopes, but not carbohydrate epitopes. Accordingly, we also failed to detect ACE2 on these sections with a polyclonal anti-ACE2 antibody (data not shown). Nonetheless, the binding ability of the RBD-Fc protein to recognize ACE2 was further assessed using HEK-293 cells transfected to express human ACE2. In flow cytometry experiments, these cells were strongly labeled by the RBD-Fc protein, unlike untransfected control cells, indicating that the protein’s binding ability to ACE2 on cell surfaces was fully preserved (Figure 3D). Thus, as we documented the functional ability of the RDB-Fc protein to bind ACE2 in a native state and as histo-blood group antigens, by contrast, are well preserved on paraffin embedded tissue sections despite the processing, collectively, these data indicate that even if the RBD of the SARS-CoV-2 spike protein does attach to an A histo-blood group antigen, this binding is very weak and difficult to detect, raising questions about its relevance to the infection process.

## Discussion

Hospital staff members have been exposed to a particularly high risk of COVID-19 due to their occupations (Jin et al., 2021). With our questionnaire, we were able to recruit over 300 couples with known ABO blood groups including at least one PCR-confirmed case of COVID-19. The frequencies of ABO phenotypes in this group were similar to those in the French general population. Likewise, the rate of secondary transmission was very similar to that estimated for spouses in a large meta-analysis of household secondary attack rates (39 vs. 38%; Madewell et al., 2020), indicating that there was no major bias among respondents. Only three secondary cases of COVID-19 were observed in the 35 couples excluded from the analysis due to index case being asymptomatic, corresponding to an attack rate of 8.6% and confirming that transmission rates from asymptomatic cases are lower than those from symptomatic cases (Madewell et al., 2020). All three cases of transmission in the excluded couples occurred in a context of ABO compatibility. The primary aim of the study was to compare secondary attack rates according to ABO compatibility between index cases and their partners. The clear identification of index cases and knowledge of the ABO blood groups of both partners made it possible to determine whether transmission occurred in a context of ABO compatibility or incompatibility. The SAR for ABO-incompatible couples was 41% lower than that in ABO-compatible couples (27.9% vs. 47.2%). Moreover, the SAR was lower for ABO-incompatible than for ABO-compatible couples regardless of the ABO blood group of the second (non-index case) partner. These observations clearly indicate that the risk of disease transmission is much lower in the presence of anti-ABO antibodies, consistent with the ABO incompatibility-dependence hypothesis. Conversely, ABO-dependent intrinsic susceptibility is unlikely to play a major role because, in ABO-compatible couples, this mechanism would result in higher secondary attack rates for blood groups A, AB and, possibly, B, than for blood group O, and no such pattern was observed. We evaluated this potential mechanism further, by testing the binding of SARS-CoV-2 RBD to synthetic or natural blood group A structures since a study reported binding of the RBD to the A type 1 tetrasaccharide using glycan microarrays (Wu et al., 2021). No signal above background was detected using the same tetrasaccharide, saliva mucins that contain A antigens based on both type 1 and type 2 backbones, or lung tissue sections of a blood group A donor that contain the A blood group antigen in all native forms. The difference between our results and those of Wu et al. may be due to the unnatural presentation of the carbohydrate structures on glycan microarrays or to a lack of sensitivity of our assays. Regardless, our negative results strongly suggest that the virus does not bind to a blood group-related carbohydrate or that, if it does, this binding is very weak and unlikely to be of any great importance.

Overall, our observations can account for the more frequent occurrence of partial protection in blood group O individuals than in blood group A and B individuals, based on the frequencies of both anti-A and anti-B antibodies. Due to the higher frequency of blood group A than of blood groups B and AB, group A individuals seldom encounter incompatible infected individuals in a population of Western European descent. This probably explains why previous cohort and case–control studies have reported individuals of blood group A to be at higher risk, and individuals of blood group O to be of lower risk of COVID-19. Blood group B is relatively rare in France (<10%), so people with this blood type encounter incompatible individuals (A + AB) frequently, accounting for the non-significant difference or slightly lower risk of COVID-19 relative to that of the other blood groups in published case–control and cohort studied. Blood group AB individuals lack both anti-A and anti-B antibodies, and had the highest SAR (54%). As blood group AB is always the rarest, the associated increase in the risk of COVID-19 passed largely unnoticed in the previous studies (Franchini et al., 2021; Goel et al., 2021; Le Pendu et al., 2021). In geographical areas where blood group A is less frequent and conversely, blood group B is more frequent, one might expect that the latter, as well as blood group AB, appear at a higher risk of COVID-19 in epidemiological studies. Indeed, this has been observed in several studies originating from India, Pakistan, Bahrain, Saudi-Arabia, and Iran (Abdollahi et al., 2020; Aljanobi et al., 2020; Almahdi et al., 2020; Padhi et al., 2020; Rahim et al., 2021; Singh et al., 2021).

In the French population, 34% of all encounters are ABO-incompatible. We found that the risk of COVID-19 transmission was 41% lower in such situations. We can therefore estimate that at least 14% of possible cases of COVID-19 transmission, at population level, were prevented by ABO incompatibility. Mathematical modeling has indicated that ABO interference would contribute to a decrease in the R_0_ coefficient of transmission (Ellis, 2021), suggesting that the overall impact of ABO polymorphism might have been higher, given the subsequent slowing of the epidemic. In Asian countries, where the frequencies of blood groups A and B are similar and blood group O is less frequent, incompatible encounters are more frequent. More individuals may therefore have benefited from the partial protection conferred by anti-ABO antibodies. Conversely, in geographic areas in which blood group O is largely dominant, ABO incompatibility would be expected to provide less protection at population level, possibly contributing to the high attack rates observed in some South American countries (Ellis, 2021; Le Pendu et al., 2021).

This study has several limitations. The hospital staff members who completed the questionnaire may not be representative of the general population. However, if any bias was introduced, it is unlikely to compromise the conclusions because we analyzed what happened in couples, regardless of other possible differences between individuals that might affect the risk of being infected and becoming ill. The only relevant parameter in the analysis is whether ABO compatibility/incompatibility affected the direction of potential transmission. We found no evidence for the existence of an ABO-dependent intrinsic susceptibility. Nevertheless, we cannot rule out that such susceptibility makes a small contribution to the higher risk of COVID-19 experienced by blood group A individuals relative to individuals of the other ABO blood groups. We also had no information about disease severity. It might be interesting, in future studies, to determine whether ABO-incompatible transmission, when it does occur, is associated with milder forms of the disease, possibly due to lower infectious doses. We also had no information about anti-A and anti-B antibody titers. The levels of these antibodies vary considerably between individuals of the same blood type (Berséus et al., 2013), and the corresponding immunoglobulin subclasses vary between the A, B, and O blood groups (Daga et al., 2021). Differences in virus neutralization efficacy might therefore be expected in conditions of ABO incompatibility. The ACE2-dependent cell adhesion of SARS-CoV mediated by the viral spike protein expressing blood group A epitopes has been reported to be blocked, in a dose-dependent manner, by anti-A antibodies (Guillon et al., 2008). The anti-A and anti-B antibody levels of individuals recently infected with SARS-CoV-2 infected individuals have been shown to be lower than those of control subjects, suggesting that ABO incompatibility-dependent protection may be dose-dependent (Deleers et al., 2021).

In conclusion, this analysis of secondary attack rates in ABO-incompatible couples found no evidence for ABO-dependent intrinsic susceptibility, consistent with the lack of evidence for SARS-CoV-2 RBD binding to blood group A (and B) antigens. By contrast, we observed that transmission in a context of ABO incompatibility was associated with a much lower SAR than transmission in a context of ABO compatibility. These observations suggest that natural anti-ABO antibodies may provide up to ≈40% protection against COVID-19 transmission, probably preventing a substantial number of cases at population level.

## Data Availability Statement

The raw data supporting the conclusions of this article will be made available by the authors, without undue reservation.

## Ethics Statement

Ethical review and approval was not required for the study on human participants in accordance with the local legislation and institutional requirements. Written informed consent for participation was not required for this study in accordance with the national legislation and the institutional requirements.

## Author Contributions

RB, AD, NR-C, and JP designed the study and the questionnaire. JP designed the experiments and analyzed the results. AB and JJ performed the experiments. SM, AC, CN, ID-H, FP, and DM made the questionnaire available to volunteers in their respective hospitals. VS contributed to the methodology and performed statistical analyses. JP wrote the manuscript. All authors contributed to the article and approved the submitted version.

## Funding

This study was supported by internal funding from Inserm, the University of Nantes and Nantes University Hospital (CHU de Nantes) and by a grant from the Région des Pays de la Loire, PROBIOSAC.

## Conflict of Interest

The authors declare that the research was conducted in the absence of any commercial or financial relationships that could be construed as a potential conflict of interest.

## Publisher’s Note

All claims expressed in this article are solely those of the authors and do not necessarily represent those of their affiliated organizations, or those of the publisher, the editors and the reviewers. Any product that may be evaluated in this article, or claim that may be made by its manufacturer, is not guaranteed or endorsed by the publisher.

## Footnotes

^1^ https://fr.statista.com/statistiques/656008/groupes-sanguins-repartition-abofrance/
